# Utility of *Borrelia*-specific IgM and IgG antibody titer determinations during a 12-year period – results from a clinical laboratory in Northern Sweden

**DOI:** 10.3389/fcimb.2023.1192038

**Published:** 2023-07-03

**Authors:** Xijia Liu, Nazanin Tabibzada, Helena Lindgren, Anders Sjöstedt

**Affiliations:** ^1^ Umeå School of Business, Economics and Statistics, Statistics, Västerbotten, Sweden; ^2^ Department of Clinical Microbiology, Umeå University, Umeå, Sweden

**Keywords:** lyme borreliosis, serological response, IgM, IgG, kinetics 2

## Abstract

Interpretation of serological findings in suspected Lyme borreliosis (LB) is challenging and IgM reactivities may have low predictive value. Therefore, if used indiscriminately, there is a risk for incorrect diagnosis of LB. To evaluate the usefulness of IgM titer determination, we performed a study of the prevalence of *Borrelia*-specific antibodies in serological samples from patients with suspected LB analyzed during the period 2010 - 2021 at the University Hospital of Umeå in Sweden. In total, 19,335 samples had been analyzed for the presence of IgG and IgM antibodies. Overall, there were higher percentages of IgM positive or borderline titers, 1,847 (9.6%) and 905 (4.7%), respectively, than IgG positive or borderline titers, 959 (5.0%) and 406 (2.1%), respectively. Peak number of samples were recorded 2012 - 2013, exceeding 1,800, whereas there were around 1,200 during 2020 - 2021. The peak number of positive IgG and/or positive IgM samples were observed during the period 2015 - 2017 with close to, or above 400, and concomitantly, the proportion of IgG positive samples increased markedly. For IgG positive samples, the increase followed a positive linear time trend (*P*< 0.001). Peak monthly numbers were observed during August, September, and October. This seasonal increase was significant for the IgG positive group (*P*< 0.05), but not for the IgM positive/IgG negative group. Repeated samples were obtained from 3,188 individuals and of the initial samples 2,817 were (88%) IgG negative and 2,315 (72%) were IgM negative and of these, 130 (4%) showed IgG seroconversion and 300 (9%) IgM seroconversion. Collectively, the data demonstrate that IgG and/or IgM positive samples represented a minority of all samples, even when repeated sampling had occurred, and IgM positive samples were much more common than IgG positive samples. Thus, the accuracy of the clinical suspicion was low and this will lead to a low predictive value of the analysis, in particular of IgM. These findings question the use of IgM titer determination as a routine analysis.

## Introduction

Lyme Borreliosis (LB) is a tick-borne disease caused by spirochetes belonging to the genus *Borrelia* (Steere et al., 2016). LB is a multi-organ disease and the most common tick-borne infection in Europe and North America (Stanek et al., 2011; Steere et al., 2016; Dessau et al., 2018; Dong et al., 2022). There are distinct clinical presentations of LB depending on the geographical localization, since the *Borrelia* genospecies differ between Europe and North America (Steere et al., 2016). In Europe, the clinical manifestations include the early stage erythema migrans (EM) and the later stages of neuroborreliosis, arthritis, and acrodermatitis chronica atrophicans. The basis for the diagnosis of LB is serology and it is routinely performed by use of enzyme immunoassays. Many reference laboratories use a two-tiered strategy, using Western blot analysis for confirmation of specificity (Smittskyddsinstitutet, 2013; Kodym et al., 2018; Lager et al., 2019; Joyner et al., 2022).

The typical serological response during LB is the appearance of antibodies within 6-8 weeks after exposure and, as for other infections, IgM antibodies are produced earlier than IgG antibodies (Stanek et al., 2011; Smittskyddsinstitutet, 2013; Lager et al., 2019). Thus, the utility of including IgM analysis in the diagnostic procedures is the earlier appearance of the antibody than IgG. Studies have shown, however, that only about 50% of patients with a duration of illness< 6 weeks are seropositive (Cutler et al., 2017; Dessau et al., 2018; Hillerdal and Henningsson, 2021). Therefore, patients with an early stage, typical EM, should be diagnosed and treated based on the clinical symptoms only (Stanek et al., 2011; Dessau et al., 2018). Since the antibody levels may remain high for months or years after infection, the predictive value of a positive IgM titer is low and very much dependent on the pre-test probability (European Centre for Disease Prevention and Control, 2016; Dessau et al., 2018; Lager et al., 2019). In addition, IgM antibody levels may be elevated due to cross-reactions with other infections, *e.g*., Epstein-Barr virus, cytomegalovirus, human immunodeficiency virus and other less defined conditions (Busson et al., 2012). Thus, current European guidelines require that clear-cut case definitions are fulfilled that corroborate the suspicion of LB before serological analysis is performed in order to achieve an acceptable predictive value (Stanek et al., 2011). Despite the guidelines, there is extensive evidence that there is frequent overuse of laboratory testing for *Borrelia*-specific antibodies, which thereby causes a high false positive rate (Dessau et al., 2010; Dessau et al., 2018; Vreugdenhil et al., 2020; Hillerdal and Henningsson, 2021). An additional drawback of IgM testing is that a negative result may be interpreted such that it excludes LB, thereby leading to underdiagnosis (Vreugdenhil et al., 2020; Hillerdal and Henningsson, 2021). Generally, IgG antibodies are not present during the first stage of infection, however, their utility is high during the later stages of LB with a positive predictive value of over 80% (Dessau et al., 2018).

The total number of *Borrelia* cases in Sweden is not exactly known since LB is not a reportable disease. However, it is estimated that there are between 5,000 and 10,000 annual cases (Läkemedelsverket, 2009). In Southern Sweden, the mean annual incidence rate of EM has been estimated to be around 460 per 100,000 (Bennet et al., 2006), but it is unknown in other parts of Sweden. There are very significant geographical differences in the seroprevalence and in endemic areas a seroprevalence as high as 29% has been reported (Gustafson et al., 1993). In the Northern part of Sweden, *Borrelia*-infected ticks generally are not as common as in other parts of Sweden and the seroprevalence is therefore presumed to be lower than those of other parts of Sweden, although comprehensive seroprevalence studies have not been performed in this part of Sweden (Wilhelmsson et al., 2013). For example, 2% of blood donors from a region in Northern Sweden were seropositive in 1990 (Gustafson et al., 1993). However, it has been noted that the prevalence in ticks does not fully explain regional seroprevalences in Sweden (Wilhelmsson et al., 2013). One explanation may be that individuals become infected in regions where they do not reside, thus, cases will be reported from regions regardless of the risk of local exposure to *Borrelia*.

The present study analyzed the presence of IgG and IgM antibodies in samples from patients with clinical suspicion of LB referred to a clinical laboratory in Northern Sweden. A question of special relevance in this regard is to understand the utility of IgM testing in a non-endemic area, due to the well-known shortcomings of the test.

## Materials and methods

### Data collection

The study was a retrospective analysis and involved all serum samples analyzed for the presence of *Borrelia*-specific antibodies at the University Hospital of Umeå during 2010 - 2021, the period for which there are electronic records available. Serum samples were received from the regions of Västerbotten, Norrbotten and Jämtland-Härjedalen. The regions had a total population of approximately 656,000 at the end of 2021 and approximately 21,000 fewer in 2010. The total number of samples was 19,335 and the data was retrieved from the laboratory system CGM Analytix (CompuGroup Medical Sweden, Solna, Sweden). The results were classified as positive, negative, or borderline depending on the specific antibody titers. In addition, information was available regarding the absolute levels of each antibody titer, the patient’s age at the time of sampling, the gender, and the sample collection date.

### Serological assays

The assays used for analysis were from 2010 until 2019, the Borrelia Select ELISA kits (Euroimmun) based on a recombinant, dimeric OspC for IgM detection and recombinant VlsE for IgG detection. From 2020 and onwards, the chemiluminescence immunoassays Liaison Borrelia IgM Quant and Liaison Borrelia IgG (Diasorin, Saluggia, Italy) was used. The assays are based on the VlsE and OspC antigens for measuring IgM reactivity and the VlsE antigen for IgG reactivity. The cut-off levels were as recommended by the manufacturers. For the Borrelia Select ELISA, positive values were >22.0 RU/ml and the borderline values 16.0 -22.0 RU/ml for both IgG and IgM, whereas for the Liaison Borrelia, the borderline values were 10.0 – 15.0 AU/ml for IgG and 18.0 – 22.0 AU/ml for IgM and the positive values were >15.0 AU/ml for IgG and > 22.0 AU/ml for IgM. According to the manufacturer, for healthy donors, the diagnostic specificity of the Liaison Borrelia IgM Quant assay is 96.6% (95% CI: 90.4-99.3%) and of the Liaison Borrelia IgG 98.0% (95% CI: 93.0-100%), The diagnostic specificity for healthy donors of the Borrelia Select IgM is 96.3% and of the Borrelia Select IgG 100%. No two-tier testing was used during the period. In Sweden, only national reference laboratories perform two-tier testing and the lack of thereof has been considered as a sufficient diagnostic measure for other laboratories.

### Statistical analysis

The non-parametric tests Spearman’s Rho and Kendall’s Tau were used to estimate if there was any association between the occurrence of IgM positive/IgG negative samples and age or gender. To calculate whether there were any significant monthly changes in the occurrence of positive samples, the proportion was calculated for each of the groups: IgM positive/IgG negative, IgG positive/IgM negative, and IgM positive/IgG positive. In order to simplify the comparisons, titers that demonstrated borderline titers were also included in each positive group. The monthly values were divided into three groups; January to June, July to August, and September to December. Two dummy variables were applied and the following regression model was applied:


$$y=\;\beta_{0}+\beta_{1}t+\beta_{2}d_{1}+\beta_{3}d_{1}t+\beta_{4}d_{2}+\beta_{5}d_{2}t$$

where y is the number of cases, t is the time variable, “month”, $d_{1}$ and $d_{2}$ is the dummy variable, if $d_{1}=1$ indicates the time period from September to December; if $d_{2}=1$ indicates the time period from January to June. This model can be viewed as a piecewise linear time trend model, coefficients $\beta_{1}$ , $\beta_{3}$ , and $\beta_{5}$ are the corresponding time trend effects in each of the three groups.

Regarding the annual data, since the piecewise linear trends pattern could not be assumed, and a global time trend was more reasonable, we fitted a linear model and a quadratic model to each type of results and identified the best goodness of fit using the following equation:


$$y=\;\beta_{0}+\beta_{1}t$$

$$y=\;\beta_{0}+\beta_{1}t+\beta_{2}t^{2}$$

where y is the number of cases of each group and t is the time variable, years.

### Studies involving human subjects

The Ethics Review Board approved the research identified in the application, 2023-02659-01.

## Results

### IgG and IgM reactivity

**Figure 1:**
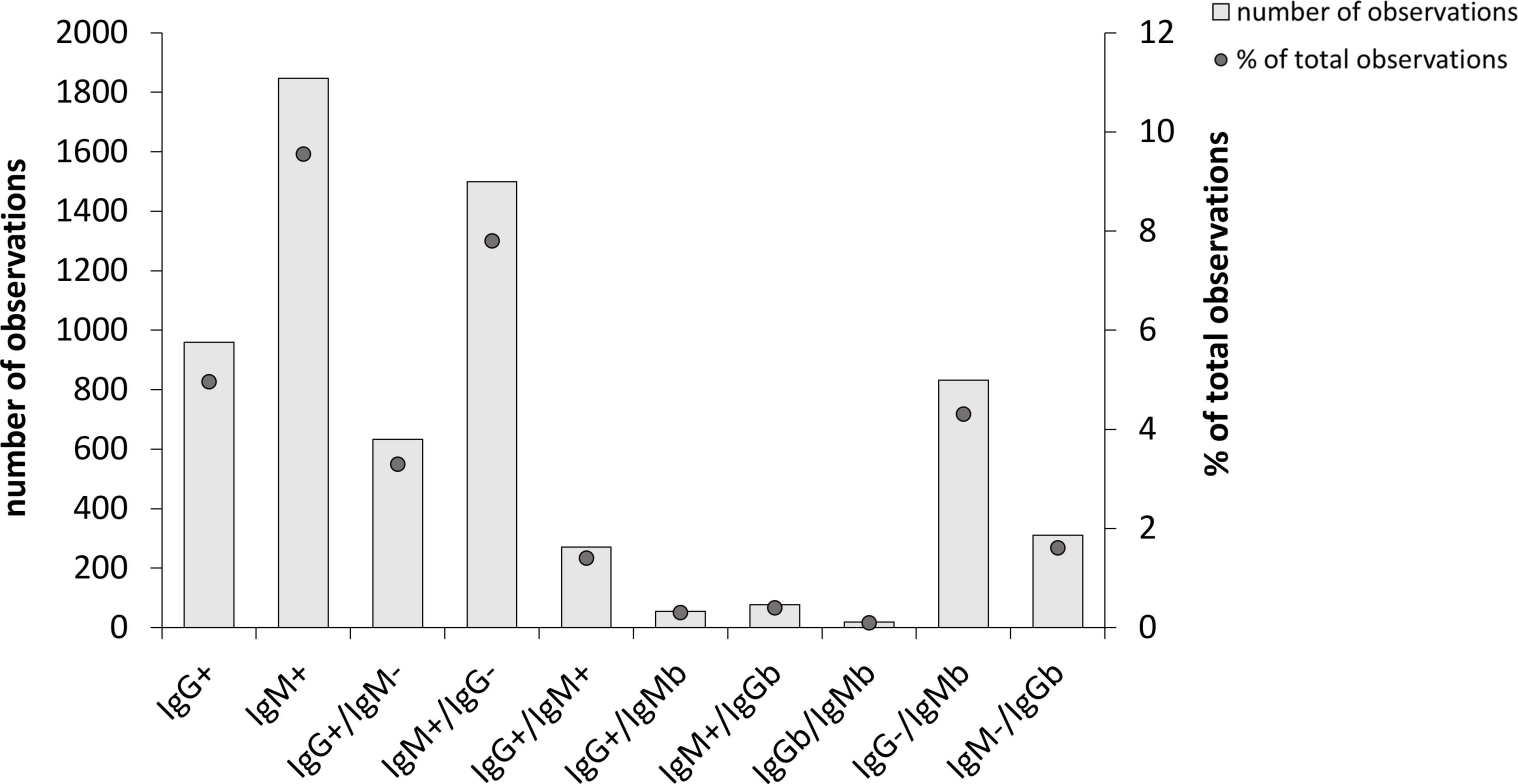
Overall *Borrelia*-antibody reactivity of 19,328 samples analyzed during the period 2010-2021. Total number of observations in each group are shown as bars and the percentage of each group relative to the total number of samples are shown as dots. 15,632 of the samples showed no antibody reactivity and are therefore not included. IgMb and IgGb indicate borderline values.

A large majority of the sera demonstrated no significant IgG or IgM titers, 15,632 out of the total of 19,328, *i.e*., 80.9% (**Figure 1**). Among the remaining samples, sera more often showed positive or borderline IgM titers than corresponding IgG titers, 1,847 and 905, vs. 959 and 406, respectively (**Figure 1**). This represented 9.6% and 7.7% *vs*. 5.0% and 2.1% of all samples. Thus, of all samples with borderline or positive titers, a majority, 71.5%, showed IgM reactivity.

Of the sera with a positive IgG titer, 34.0% showed a positive or borderline IgM titer. When IgG titers were at borderline levels, the likelihood of a significant IgM titer was low, since 19.0% of the sera were IgM positive and 4.4% had borderline values.

Sera with positive IgM titers showed low likelihood of positive or borderline IgG titers, since only 18.9% showed a significant IgG titer. A serum with an IgM borderline titer was less likely to display a significant IgG titer, since only 8.1% of the samples demonstrated an IgG positive or borderline titer.

### Influence of age and gender on IgM titers

In view of the substantial proportion of samples that were IgG negative and IgM positive or borderline, we analyzed these 2,331 samples further and asked whether gender or age was an important parameter that increased the likelihood of this result, as demonstrated in other investigations (Strle and Stanek, 2009). Both Spearman’s rho and Kendall’s tau were utilized and gave very similar *P* values and therefore only the former values are shown (**Table 1**). We observed that the parameters male and > 65 years of age affected the results, albeit non-significantly, since *P* values were between 0.05 and 0.07 (**Table 1**). The other parameters did not affect the likelihood of the result IgG negative and IgM positive or borderline titer (*P* > 0.27).

**Table 1:** Effects of gender and age on the occurrence of IgM positive/IgG negative samples^1^.

	Male	Female	0-30	31-50	51-65	>65
IgG^-^/IgM^+^	0.127^2^ (0.051)^3^	0.027 (0.555)	0.078 (0.270)	0.045 (0.505)	-0.007 (0.989)	0.165 (0.067)

^1^The proportion of IgM positive/IgG negative samples in each gender group and age group was compared to the proportion of all IgM positive/IgG negative samples (2,331/19,328) and the effects of age and gender were analyzed by Spearman's Rho and Kendall’s Tau.

^2^Spearman’s rho coefficient.

^3^P value based on Spearman’s rho coefficient.

### Analysis of repeated sampling

Repeated samples were obtained from 3,188 individuals and these results were further analyzed. Samples were obtained from a small number of individuals more than twice, but these were too few to provide any useful statistical data, therefore the analysis was based on the first two samples obtained. The initial samples were predominantly IgG negative, 2,817 (88%), or IgM negative, 2,315 (72%) (**Table 2**). Upon repeated sampling of individuals with an initially negative sample, 130 (4%) became IgG borderline or positive and 300 (9%) became IgM borderline or positive. Among the initial samples, 89 (3%) were IgG borderline and of these, 30 were IgG negative and 20 IgG positive upon repeated sampling (**Table 2**). 235 (7%) of the initial samples were IgM borderline and of these, 45 became IgM negative and 88 IgM positive upon repeated sampling. Among the remaining samples, 282 (9%) were initially IgG positive and 638 (20%) IgM positive. Of these, 24 were IgG negative and 61 IgM negative, respectively, upon repeated sampling (**Table 2**). Thus, also a large majority of the repeated samples showed no seroreactivity, since 2,741 (86%) were IgG negative and 2,121 (66%) IgM negative (**Table 1**).

**Table 2:** Results of repeated sampling for *Borrelia*-reactivity^1^.

Original result	Repeated IgG result			Repeated IgM result			
	negative	borderline	positive	negative	borderline	positive	
negative	2,687 (84%)	59 (2%)	71 (2%)	2,015 (63%)***^2^	143 (4%)***	157 (5%)***	
borderline	30 (1%)	39 (1%)	20 (1%)	45 (1%)**	102 (3%)	88 (3%)*	
positive	24 (1%)	22 (1%)	236 (7%)	61 (2%)	72 (2%)	505 (16%)	

^1^Repeated samples were obtained from 3,188 individuals, of which 2,817 (88%) were initially IgG negative, 89 IgG borderline, and 282 IgG positive. 2,315 (73%) samples were initially IgM negative, 235 IgM borderline, and 638 IgM positive. Upon repeated sampling from the same individuals, 2,741 (86%) were IgG negative and 2,121 (67%) IgM negative.

^2^Indicates pairwise comparison to assess the likelihood of the indicated conversion of IgG vs. IgM as determined by the Chi square text. For example, the P value in the box “borderline→negative” indicates that there was a significantly different likelihood of this conversion between IgG and IgM. *P < 0.05; **P < 0.01; ***P < 0.001.

The median time between sampling was 60 days and 60% were sampled within the first 100 days. Among the remaining, a minority of samples were taken more than one year apart, thus, they likely did not represent follow-up samples.

The results demonstrate that although there existed a clinical suspicion of LB, the likelihood of serological confirmation was low, since even after repeated sampling, a large majority was IgG negative and/or IgM negative.

### Annual and seasonal variations in *Borrelia* sampling and antibody reactivity

**Figure 2:**
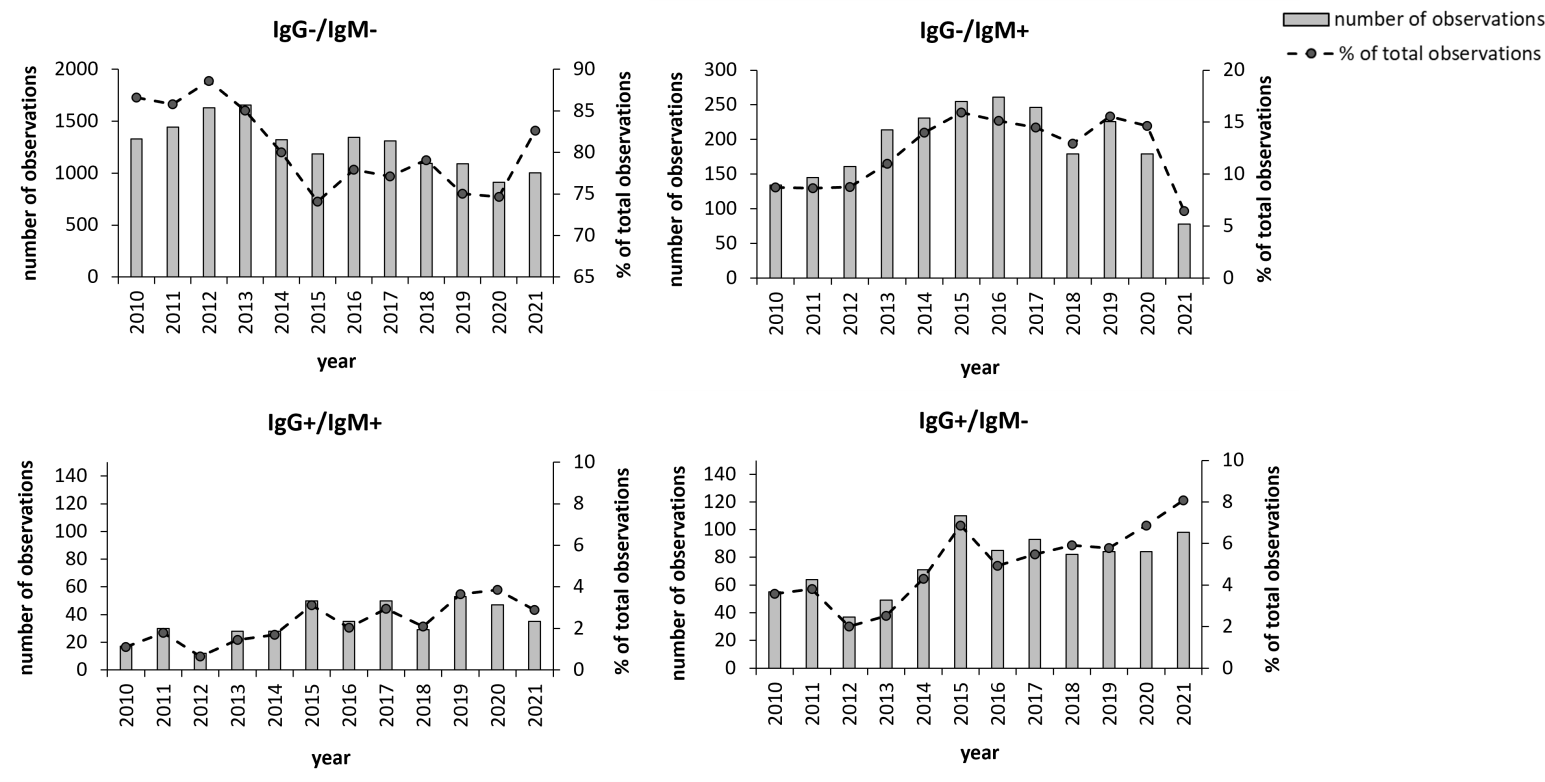
Annual distribution of the 19,328 samples during the period 2010 - 2021. Total number annual samples are shown as bars and the annual percentage of samples relative to the total number of samples are shown as dots. The groups IgG^+^ and IgM^+^ also include the samples with borderline values for the respective antibody class. *P < 0.05; **P < 0.001; ***P < 0.001.

We also analyzed how the sampling varied seasonally and annually during the 12 years. The peak number of samples were recorded 2012 and 2013 with more than 1,800 and 1,900, respectively (**Figure 2**). During the period 2014 - 2019, there were fewer total samples, between 1,400 and 1,700 and during 2020 and 2021, the numbers were distinctly lower, around 1,200. However, despite the decreasing total number of samples, the peak number of positive IgG and/or positive IgM samples was observed during the period 2015 - 2017 with close to, or above 400 total positive samples, *i.e*., around 25% (**Figures 2B–D**). The number of IgG-positive samples increased markedly 2015 to 160 and during the period 2015 – 2021, the number of IgG positive samples varied between 130 to 160, whereas they varied from 50 to 100 between 2010 to 2014 (**Figures 2C, D**). Thus, the percentage of IgG-positive samples increased markedly during the period 2015 - 2021 compared to the preceding period, 8.9% *vs*. 4.6%.

**Figure 3:**
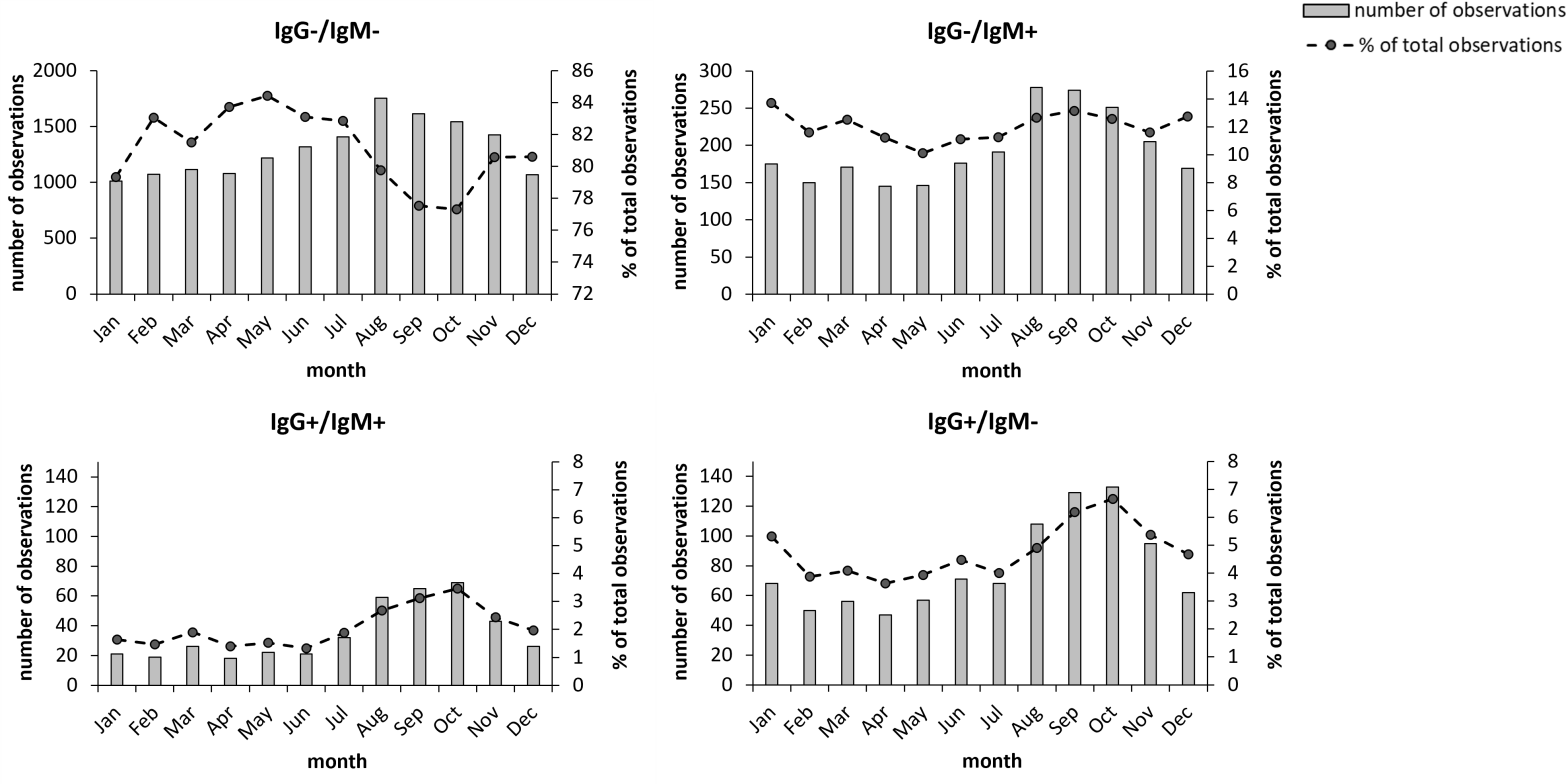
Monthly distribution of the 19,328 samples during the period 2010-2021. Total number monthly samples are shown as bars and the monthly percentage of samples relative to the total number of samples are shown as dots. The groups IgG^+^ and IgM^+^ also include the samples with borderline values for the respective antibody class. Bold numbers indicate significant differences (*P* < 0.05).

There was clear seasonal variation in the sampling. During the period December to April, the total numbers were lowest, between 1,500 and 1,700, then increased during summer and reached a peak in August, September, and October, when numbers ranged from 2,450 to 2,650 (**Figures 3A–D**). The number of IgM and/or IgG positive samples varied between 210 - 290 during the period December to July and then increased to around 450 in August, September, and October and were also increased during November (**Figures 3B–D**). This seasonal trend was similar for the two groups of IgG positive samples with increasing numbers during the period August to November (**Figures 3C, D**), but not for the IgM positive/IgG negative group (**Figure 3B**). The percentage of IgG-positive samples during the period August to November was 8.7% *vs*. 5.9% for the other part of the year.

To assess whether the aforementioned changes were statistically significant, modeling of the variations was performed. By analyzing the annual data, it was observed that the group IgM positive/IgG negative demonstrated a significant quadratic time trend, *i.e*., an increasing trend followed by a decrease (**Figure 2B**), whereas the other two groups fitted a linear model (**Figures 2C, D**). We next examined the regression coefficients and whether there were significant time trends for each group. For each of the groups IgG positive/IgM negative and IgG positive/IgM positive, a linear time trend model was sufficient to capture the time trend effect, and there was a significant positive effect with a coefficient of time trend of 0.002 (*P*< 0.001) and 0.004 (*P*< 0.000), respectively. For the group IgM positive/IgG negative, a quadratic time trend was the best fit, and the coefficients were 0.032 for t (*P*< 0.003) and -0.002 for t^2 (*P*< 0.005). Thus, the number of cases differed significantly between years for each group, however, the group IgM positive/IgG negative did not show the same trend as did the IgG positive groups.

With regard to seasonal effects, after fitting the model, t tests were performed on coefficients β1 (July-August), β3 (September-December), and β5 (January – June). If a coefficient is significantly different from 0, then there is a change between months for the corresponding period. The results are presented in **Table 3**. Each of the three groups demonstrated a significant decreasing trend for the period January-June, whereas only the groups IgG positive/IgM negative and IgG positive/IgM positive showed significant increases during the period July – August. During the period September – December, the groups IgG positive/IgM negative and IgG positive/IgM positive, but not the IgM positive/IgG negative group, showed a significant decreasing trend. Thus, also the seasonal trends of the IgM positive/IgG negative group differed from those of the IgG positive groups.

**Table 3:** Analysis of the seasonal variation in the occurrence of different types of *Borrelia*-reactivity^1^.

Group	*β_1_*	*P* value	*β*_3_	*P* value	*β*_5_	*P* value
IgG^+^/IgM^+^	0.006	**0.004**	-0.013	**0.000**	-0.006	**0.004**
IgG^+^/IgM^-^	0.011	**0.025**	-0.021	**0.007**	-0.012	**0.020**
IgG^-^/IgM^+^	0.009	0.135	-0.008	0.312	-0.015	**0.042**

^1^β1 represents the time trend effect during the period July-August, β3 represents the time trend effect during the period September-December, and β5 represents the time trend effect during the period January–June. All analyses were based on the data presented in **Figure 2**. Bold numbers indicate significant differences (P < 0.05).

## Discussion

The utility of serology for the diagnosis of LB has been much studied and it is well-known that the frequent use of the diagnostic measure for EM patients is a problem (Dessau et al., 2018; Strizova et al., 2020; Vreugdenhil et al., 2020; Hillerdal and Henningsson, 2021; Joyner et al., 2022). Meta-studies have estimated that the sensitivity is very low, in the range of 29 - 49% (European Centre for Disease Prevention and Control, 2016), due to the fact that seroconversion occurs late in LB and is often preceded by EM. The specificity is estimated to be in the range of 90 – 95%, but overall, the positive predictive value of the test is very low and, thus, testing does not lead to any value for validation of the EM diagnosis beyond that of the clinical presentation *per se* (European Centre for Disease Prevention and Control, 2016; Dessau et al., 2018). Therefore, there are strict guidelines to ensure that the serology is used in such a way that the results can be rationally interpreted (Stanek et al., 2011). However, in Sweden, as well as in other European countries, the adherence to the guidelines is low and it has been estimated that only 20-30% of the serological samples are taken according to current guidelines in patients with positive serology (Dessau et al., 2018; Vreugdenhil et al., 2020; Hillerdal and Henningsson, 2021). There are no comprehensive studies on individuals with negative serology, but it is reasonable to assume that the percentage in this group sampled according to the guidelines is even lower. It has also been argued that one problem could be a lack of understanding on behalf of the clinicians when interpreting the serological responses (Vreugdenhil et al., 2020; Hillerdal and Henningsson, 2021). For example, based on information from patient journals, it was concluded that many physicians believe that an IgM response is a prerequisite for an existing infection and that the lack thereof negates an active infection (Hillerdal and Henningsson, 2021). Such lack of understanding will lead to unnecessary antibiotic treatment, but also to lack of treatment for some LB patients.

Our study identified that a large majority of the sera referred to the clinical laboratory with suspected LB, 80.9%, demonstrated no significant IgG or IgM titers. Among those with significant titers, 34.0% of the sera with a positive IgG titer showed a positive or borderline IgM titer, whereas 18.9% of sera with a positive IgM titer showed a positive or borderline IgG titer. We believe this is logical, since IgG titers occur during the later stages of LB, but sampling is likely not performed during very late stage since patients have had symptoms for quite a long time and therefore already contacted the health care system. At this time, IgM titers may still persist. When IgM titers were positive, a rather low proportion was also IgG positive, presumably most of these samples represent early stage EM, when IgM, but not IgG seroconversion had occurred, but some may also represent false IgM positive samples. Overall, positive IgM titers were much more frequent than positive IgG titers, since IgM positive or borderline titers represented 17.3% of all samples, whereas the corresponding IgG titers represented 7.1% of all samples. Thus, samples with IgM borderline or positive titers represented 71.5% of all reactive samples.

Analysis of repeated sampling demonstrated that although there existed a clinical suspicion of LB, the likelihood of serological confirmation was low, since of the initial samples, 88% were IgG negative and 72% IgM-negative and of these, only 4% showed IgG seroconversion and 9% IgM seroconversion. Collectively, the data demonstrate that the proportion of IgG or IgM positive samples represented a minority of all samples, even when repeated sampling had occurred. Consistently, IgM positive samples were much more common than IgG positive samples. Both observations, *i.e*., the very low overall proportion of positive samples and low degree of seroconversion, demonstrate that the accuracy of the clinical suspicion was low and this will markedly affect the predictive value of the analysis. In particular, considering the predominance of IgM positive samples, the predictive value, of an IgM positive sample can be assumed to be low and this questions the routine that analysis of IgM is performed on all sera.

Results from a Swedish study based on data from Jönköping are of relevance since the results of all *Borrelia* serology testing performed during one year were analyzed (Hillerdal and Henningsson, 2021). It was found that isolated IgM positivity was quite rare, representing only 1.0% of all samples. Even when the samples positive for both IgM and IgG were included, the total number of IgM positive samples represented less than 3.1%. Notably, the two groups combined represented 17.2% of all samples in the present analysis. Of all Jönköping samples, 12.8% demonstrated positive IgG titers, with or without IgM titers, whereas the corresponding figure for the Umeå samples was 7.1%. Thus, the results are very discrepant, since samples positive for IgM represented 71.5% of all samples with positive titers in the present study, whereas the corresponding percentage for Jönköping was 22.3%. There are obvious seroepidemiological differences between the two regions and the seroprevalence is expected to be much higher in the Jönköping region, since it is endemic for LB (Berglund et al., 1995) and this could explain the higher percentage of IgG positive samples in Jönköping. Notably, during the period 2015 - 2021, the percentage of IgG positive samples in Umeå was 8.9%, indicating that LB cases are increasing, but still not as high as in Jönköping.

The very high proportion of IgM positive Umeå samples cannot be explained by differences in the seroprevalence. It is mentioned in the publication from Jönköping that the cut-off of the assay had been increased two-fold compared to the recommendation of the manufacturer, due to the high seroprevalence in the region. This increase of the cut-off is similar to the difference in the cut-off between borderline and positive titers of the Umeå assay. Even if the borderline values are excluded, the positive IgM values of the Umeå data represented 9.6% of all samples, thus more than three times higher than the corresponding Jönköping value. The data from Jönköping did not provide details regarding the patients and therefore, we cannot determine whether such differences may explain some of the discrepancies. Our detailed analysis of the 3,118 samples that were IgG negative and IgM positive or borderline revealed that the parameters male and > 65 years of age were more common in this group, however, without being significant parameters. Age and gender have previously been reported as parameters that affect the *Borrelia*-specific serological response (Strle and Stanek, 2009). However, since these parameters did not significantly affect the results, it is highly unlikely that they would explain the large discrepancies between the data from the current analysis and the data from Jönköping. In view of the described discrepancy and the relatively low number of repeated samples that showed seroconversion, 9% for IgM and 4% for IgG, the conclusion is that IgM has a low predictive value and that a considerable proportion of the IgM reactivity represents false positive results. Previous analyses have indicated that many IgM positive samples are found in individuals with non-specific symptoms and that the titers may be false positive or simply reflect previous *Borrelia* exposure, but not active disease (Busson et al., 2012). Therefore, the data support the notion that IgM analysis should be performed only after a triage, *e.g*., in patients with distinct clinical manifestations that agree with either of the clinical forms of LB as stated in the European guidelines (Stanek et al., 2011). Such triage has been proposed previously as a result of meta-analyses (Dessau et al., 2018).

An important question is whether the exclusion of IgM titers from the routine diagnostic procedures would lead to missed or delayed diagnosis of LB. The present data cannot unambiguously answer the question, but of relevance is the repeated sampling that showed seroconversion. Since 9% showed seroconversion for IgM, whereas the corresponding number for IgG was 4%, it is possible that the difference reflects a number of LB patients that would not be identified by means of IgG testing alone. Whether triage would lead to the identification of these patients remains to be investigated. Notably, it has been observed that the use of the VlsE antigen as used here for IgG is highly sensitive and that IgM detection appears to have no significant advantage over this type of IgG analysis (Dessau et al., 2018).

Our analyses of annual and seasonal variation in sampling and positive samples identified some interesting trends. Although total sampling decreased somewhat after 2013, the number of positive samples increased both proportionally and in total numbers thereafter. We cannot explain the variation between the years, with the exception of the distinctly lower numbers during 2020 and 2021, since this very likely was explained by the pandemic. The same decreasing trend was generally observed for clinical laboratory analyses during those two years (Durant et al., 2020). Interestingly, there was a clear trend of an increasing number of positive samples, since the peak number of positive IgG and/or positive IgM samples was observed during the period 2015-2017. Moreover, the number of IgG positive samples and the proportion of IgG samples increased markedly from 2015 and onwards, approximately doubling compared to the preceding period. All evidence indicates that the increasing number of positive samples represents a true increase in the number of *Borrelia* cases, although we cannot verify it since the disease it is not reportable. It agrees with a general trend in Sweden of an increasing number of *Borrelia* cases and also field studies that indicate that the number of infected ticks is increasing in Norrland, particularly along the coast (Jaenson et al., 2012). There is also circumstantial evidence that a proportion of the cases had been infected outside Norrland, although the sampling occurred in Norrland. Interestingly, although the total number of IgG positive samples did not increase during 2020 - 2021, the percentages of IgG positive samples increased during those years. Possibly, the pandemic may have resulted in selection of patients visiting health care facilities, leading to the increased proportion of IgG positive samples.

The seasonal variation of the sampling was rather expected and mirrors the incidence of tick exposure. The number of total samples increased starting from May and peaked in August, September, and October, when total numbers and the number of positive IgG samples increased very much compared to the period December to July. Presumably, even though the peak started in August, this reflects the time before onset of a positive serological response; thus, some of the individuals may have been infected earlier in summer. Notably, the occurrence of IgM positive/IgG negative samples did not follow the same statistical seasonal trend as did the IgG positive samples, since the former group did not show any significant increase during the period August-October, in contrast to the IgG positive samples. This is a further argument that a proportion of samples of the former group represents false positive results.

The data presented herein are based on samples collected during a long period, 12 years, and therefore variations that occurred during limited periods, such as the pandemic, do not affect the overall conclusions. However, there are still limitations with regard to the data set. Notably, sera were not routinely subjected to two-tier testing, since such testing is performed by national reference laboratories in Sweden and only selected sera were therefore referred to two-tier testing. The individuals tested represent heterogenous clinical entities, since they include otherwise healthy patients with variable symptoms that may or may not be indicative of LB, but also individuals with disease, *e.g*., pathological neurological symptoms, that are routinely screened for LB. Another limitation is that only a minority of individuals was subjected to repeated sampling. Further, two different assays for determination of the serological reactivity was utilized.

In conclusion, our data indicate that the number of positive *Borrelia* samples has been increasing since 2015. Even though there is a clear trend that the increase includes IgG samples, of the total number of positive samples during the 12-year period, IgM positive samples were predominant and represented over 70%. Our data do not directly identify whether some of the IgM positive samples represent false positive samples, but provide circumstantial evidence that this is the case and therefore questions the use of IgM titer determination as a routine analysis.

## Data availability statement

The raw data supporting the conclusions of this article will be made available by the authors, without undue reservation.

## Author contributions

AS and NT designed the study. XL, HL and AS analyzed the data. XL, HL, NT and AS wrote the manuscript. All authors contributed to the article and approved the submitted version.
